# A mathematical model of HCV transmission dynamics with sex stratification and environmental effects

**DOI:** 10.1371/journal.pone.0336374

**Published:** 2025-12-01

**Authors:** Mlyashimbi Helikumi, Josiah Mushanyu, Adquate Mhlanga

**Affiliations:** 1 Mbeya University of Science and Technology, College of Science and Technical Education, Department of Mathematics and Statistics, Mbeya, Tanzania; 2 Department of Computing, Mathematical and Statistical Science, University of Namibia, Windhoek, Namibia; 3 The Program for Experimental and Theoretical Modeling, Division of Hepatology, Department of Medicine, Stritch School of Medicine, Loyola University Chicago, Maywood, Illinois, United States of America; The First Hospital of Jilin University, CHINA

## Abstract

This study primarily aims to determine how sex-specific behaviors influence Hepatitis C Virus (HCV) transmission dynamics among people who inject drugs (PWID), and to identify key parameters and interventions that most effectively reduce infection prevalence. Despite the availability of effective treatment, HCV remains a major public health challenge particularly among PWID, with sex-specific differences influencing the transmission dynamics. In this study, we developed a sex-structured deterministic mathematical model to investigate HCV transmission through contaminated needles, focusing on gender-specific patterns among PWID. Unlike previous models, our work separates transmission by sex and also captures differences between high-risk and low-risk injecting behavior through transmission and needle reuse parameters.The model classifies the population into distinct HCV related compartments for both males and females, while also incorporating an environmental pool of contaminated needles. We computed and analyzed the systems reproduction threshold and steady states, identifying conditions under which the disease persists and scenarios where backward bifurcation may occur. Sensitivity analysis identified the most influential factors on acute infection prevalence, such as rates of needle contamination, sex-specific contact behaviors, and recovery outcomes among males. Simulation results show that males experience a higher burden of acute and chronic HCV infections. Increasing the viral decay rate in needles leads to a notable decline in infections, highlighting the effectiveness of environmental interventions like needle sterilization. Additionally, reducing risky behaviors in both sexes produces the largest overall reduction in transmission, while improving needle exchange efforts by lowering the reuse of uncontaminated needles further suppresses disease spread. Our findings highlight the need for integrated harm reduction with enough, gender informed coverage, particularly ensuring sufficient reach among men, while future work should evaluate sex differential uptake and efficacy.

## 1 Introduction

Hepatitis C, caused by the hepatitis C virus (HCV), is a significant cause of liver inflammation that can lead to both acute and chronic hepatitis. The disease severity varies widely, ranging from mild illness to serious, lifelong conditions such as liver cirrhosis and cancer. HCV is primarily a bloodborne virus, transmitted through contaminated blood. Common transmission routes include unsafe injection practices, inadequate healthcare procedures, unscreened blood transfusions, injection drug use, and sexual practices involving blood exposure [1]. In the US alone, new HCV infections now exceed 30,500 annually, with the ongoing opioid epidemic contributing to recent outbreaks [2]. Between 2010 and 2015, the incidence of HCV surged by 294%, primarily due to the transition from prescription opioid use to injected heroin and the increasing number of people who inject drugs (PWID) [3,4]. The consequences of untreated HCV are significant, contributing substantially to liver-related mortality. The estimated healthcare costs associated with HCV in the USA are projected to rise from $6.5 billion to $9.1 billion annually by 2024 [5,6]. In most developed and many developing countries, PWID remain the primary risk group for HCV infection, making them a priority for prevention interventions [7]. The global burden of HCV among PWID is particularly high, with an estimated 52% of PWID having a history of HCV infection [8]. As countries strive to meet WHO elimination targets, a focused effort on preventing transmission among PWID is crucial. Despite significant advancements in HCV treatment, such as the introduction of direct-acting antivirals (DAAs) and host-targeting agents (HTAs), achieving a 100% effective HCV therapy remains a challenge. The previous standard therapy, pegylated interferon (IFN), had low efficacy and was associated with major side effects [9]. Furthermore, vaccine against HCV is currently not available.

In many countries, HCV infections are predominantly seen among people who inject drugs, leading to a significant emphasis on treating this group to reduce both prevalent and incident infections, a strategy known as, treatment-as-prevention [10]. Women make up approximately one-quarter to one-third of individuals who inject drugs [11], and the incidence of HCV is 36% higher among women compared to men in this group, attributed to gender-specific risk patterns [12,13]. While some population data indicate that women and men with HCV are starting and completing treatment at similar rates [14], there is evidence that women who inject drugs are initiating treatment at lower rates than their male counterparts [15,16]. Despite this, literature on HCV prevalence generally does not show significant differences between sexes. A brief review of longitudinal studies on HCV among people who inject drugs reveals inconsistent findings. For instance, Micallef et al. [17] found women in Sydney, Australia, were less likely than men to become infected, while Maher et al. [18] reported a higher likelihood of infection among women in a prospective study. Additionally, two prospective studies in Canada and Switzerland found no significant sex differences in infection rates [19,20]. However, reports from San Francisco, The Netherlands, and China suggest a significantly higher incidence of HCV among female people who inject drugs compared to males [21–23]. These variations underscore the need for a more systematic analysis of HCV infection differences between female and male injectors.

Many studies have analyzed HCV transmission dynamics, including among people who inject drugs (PWID). These studies address various questions and highlight different facets of the epidemic, but many overlook the aspect of gender. Some studies developed compartmental or epidemiological models of HCV transmission, emphasizing injectable behaviors and population level spread without disaggregating by sex (e.g., [24–28]). These papers typically simulate direct contact or baseline risk behaviors in settings such as urban China or the UK, identifying key transmissibility parameters while treating PWID as a homogeneous group. Other studies examined prevention and treatment interventions, including antiviral therapy, harm reduction strategies, and micro elimination efforts, and assess impacts on incidence and adherence (e.g., [29–36]). For example, some studies with models focusing on China, Chicago, and New Hampshire show that treatment can curb outbreaks, yet they often omit environmental reservoirs or relapse dynamics in gendered contexts. Some HCV studies incorporated indirect transmission mechanisms, such as through contaminated equipment or environmental factors like needle bleaching, using mathematical frameworks to highlight equipment centrality (e.g., [37,38]). These studies highlighted the role of shared needles in sustaining infections. Finally, some papers addressed specialized angles such as fibrosis progression by transmission route or fractional order dynamics of chronic infection, and they provide insights into long term complications in diverse populations (e.g., [39,40]).

All the previous studies have improved understanding of within host processes and the consequences of injection behavior, yet a notable gap remains. Gender heterogeneity, which shapes risk behaviors, access to care, and transmission patterns, is rarely incorporated. In contrast to models that aggregate sexes or treat transmission as purely contact based, our study explicitly integrates sex structure with an environmental reservoir of infected needles. To the best of our knowledge, this is the first model to examine gender heterogeneity and indirect needle mediated transmission within a unified host environment framework. Meeting these objectives is important for designing targeted strategies and interventions that account for gender specific factors. By examining the impact of gender on HCV spread in PWID populations, this study addresses a clear gap in the literature and supports more effective prevention and treatment policies. Our study draws inspiration and foundational ideas from the following manuscripts: [24,37].

The subsequent sections of this paper are organized as follows: Sect 2 outlines the model analysis, including the computation of steady states and the analysis of their stability. Sect 3 presents the results, which include the numerical simulations and the partial rank correlation coefficients (PRCC). Sect 4 provides the discussion and concluding remarks to summarize the paper.

## 2 Model description

In this manuscript, we propose a sex-structured deterministic mathematical model to explore the dynamics of HCV transmission, particularly focusing on indirect transmission through infected needles from the environment. We assume transmission occurs exclusively via parenteral exposure from contaminated needles/syringes among PWID; sexual transmission is not modeled. In this context, humans are considered the direct hosts of the virus, while the environment primarily represents the presence of needles, a key vector in the indirect transmission of HCV. Our model categorizes the human population, or hosts, into four distinct classes for each gender at any given time *t*: susceptible individuals (*S*_*i*_), those with acute HCV (*A*_*i*_), chronic HCV (*C*_*i*_), and the recovered population (*R*_*i*_), where *i* = *f* for females and *i* = *m* for males. Consequently, the total human population is represented as $N=N_{f}+N_{m}$, with $N_{i}=S_{i}+A_{i}+C_{i}+R_{i}$. We assume homogeneous mixing within and between sexes as a standard simplifying assumption, which allows model tractability in the absence of detailed network data for PWID.

In our framework, $\Lambda_{i}$ signifies the rate at which new susceptibles are recruited through birth, while $\mu_{i}$ denotes the natural mortality rate in the population. The disease-induced mortality rate is defined by parameter $v_{i}$. The model system, detailed in Eq (1) describes the dynamics of HCV transmission within this host-environment framework.

$$\frac{dS_{i}}{dt}=\Lambda_{i}-(\mu_{i}+\lambda_{i})S_{i},\;\frac{dA_{i}}{dt}=\lambda_{i}S_{i}-(\mu_{i}+\theta_{i})A_{i},\;\frac{dC_{i}}{dt}=\alpha_{i}\theta_{i}A_{i}-(\mu_{i}+v_{i})C_{i},\;\frac{dR_{i}}{dt}=(1-\alpha_{i})\theta_{i}A_{i}-\mu_{i}R_{i},\;\frac{dW_{u}}{dt}=b-(\lambda_{c}+r_{u})W_{u}+\phi W_{c},\;\frac{dW_{c}}{dt}=\lambda_{c}W_{u}-(\phi +r_{c})W_{c},\;i=m,f\;$$ (1)

with

$$\lambda_{i}=\frac{\beta_{i}W_{c}}{W_{u}+W_{c}},\;i=m,f$$ (2)

and

$$\lambda_{c}=\frac{\beta_{c}(A_{m}+A_{f}+C_{m}+C_{f})}{W_{u}+W_{c}}.$$ (3)

The rate of new host infection due to infected environmental agents *W*_*c*_ is defined by $\lambda_{i}$, and $\lambda_{c}$ quantifies the rate at which environmental agents (needles) become infected by hosts. Parameter $\beta_{i}$ signifies the strength of interaction between susceptible hosts *S* and the environmental reservoir (needles), while $\beta_{c}$ indicates the interaction strength between infected hosts ($A_{m}+A_{f}+C_{m}+C_{f}$) and the environmental reservoir. Both $\beta_{c}$ and $\beta_{i},\;i=m,f$ are generally proportional to the contact rate but differ in their directional influence on infection spread. It is important to note that the fraction $\frac{W_{u}}{W_{u}+W_{c}}$ represents the proportion of environmental agents that are currently non-infectious. Moreover, the *W*_*c*_ compartment influences the rate of infection as the fraction $\frac{W_{c}}{W_{u}+W_{c}}$. The epidemic progression is driven by interactions between the susceptible human population and the infected environmental compartment *W*_*c*_, as well as between infected individuals (*A*_*m*_, *A*_*f*_, *C*_*m*_, *C*_*f*_) and the uninfected environmental compartment *W*_*u*_. Here, *W*_*u*_ denotes the number of uncontaminated needles available in circulation, while *W*_*c*_ represents contaminated needles capable of transmitting HCV infection among PWID. Infected needles are discarded at a rate of *r*_*c*_, while uninfected needles are discarded at *r*_*u*_. We incorporate the survival of HCV on needles, as outlined in various studies [41,42], through the parameter *ϕ*, quantifying the virus decay rate on infected needles. Parameter *b* represents the introduction rate of uninfected needles. A portion $\alpha_{i}$ of individuals with acute infections progress to chronic infection at rate $\theta_{i}$, with the remainder recovering. The key model quantities described above are summarised in Table 1.

**Table 1:** Model compartments and parameters used in the HCV host–environment transmission framework.

Symbol	Type	Definition
*S*_*i*_	Compartment	Susceptible individuals (sex i=m,f)
*A*_*i*_	Compartment	Acutely infected individuals
*C*_*i*_	Compartment	Chronically infected individuals
*R*_*i*_	Compartment	Recovered individuals
*W*_*u*_	Compartment	Uninfected needles in the environment
*W*_*c*_	Compartment	Infected needles in the environment
$\Lambda_{i}$	Parameter	Recruitment rate of susceptibles
$\mu_{i}$	Parameter	Natural mortality rate
*v*_*i*_	Parameter	Disease-induced mortality rate
$\theta_{i}$	Parameter	Progression rate from acute to chronic
$\alpha_{i}$	Parameter	Proportion progressing to chronic infection
$\beta_{i}$	Parameter	Infection rate of hosts via contaminated needles
$\beta_{c}$	Parameter	Contamination rate of needles by infected hosts
*b*	Parameter	Recruitment rate of uninfected needles
*ϕ*	Parameter	Viral decay/survival rate on infected needles
*r*_*u*_	Parameter	Discard rate of uninfected needles
*r*_*c*_	Parameter	Discard rate of infected needles

## 3 Model analysis

### 3.1 Invariant region

**Theorem 3.1.** *For the model (1) to be mathematically tractable and epidemiological meaningful, it can be ascertained that the region of biological interest is well posed. Thus, we claim*


$$\Omega \;=\;\left\{\;(S_{i},A_{i},C_{i},R_{i},W_{u},W_{c})\in {\mathbb{R}}_{+}^{10}\;|\;N_{i}\leq \frac{\Lambda_{i}}{\mu_{i}},\;W_{u}+W_{c}\leq \frac{b}{\min(r_{u},r_{c})},\;i\in \{m,f\}\right\}$$

*is attracting and positively invariant with respect to model* (1).

The proof is provided in the Supplementary Material (Appendix A).

### 3.2 Reproduction number

The basic reproduction number is a key metric used to evaluate the likelihood of a disease spreading within a host population during an outbreak. This number indicates that the disease will eventually die out when it is below one, and conversely, that the disease will continue to spread when it is above one. To calculate this number for model (1), it is essential to first identify the disease-free equilibrium state. Through direct calculation, we can determine that the disease-free equilibrium for model (1) is as follows.

$${ℰ}^{0}=\left(\frac{\Lambda_{m}}{\mu_{m}},0,0,0,\frac{\Lambda_{f}}{\mu_{f}},0,0,0,\frac{b}{r_{u}},0\right).$$ (4)

To derive the reproduction number, a commonly used method is the next-generation matrix approach, as detailed in reference [43]. Following the NGM methodology, we define two matrices at the disease-free equilibrium: a non-negative matrix (*F*) representing the generation of new infection terms, and a non-singular matrix (*V*) representing other transfer terms in the system.

$$F=(\begin{array}{ccccc} 0 & 0 & 0 & 0 & \frac{\beta_{m}r_{u}\Lambda_{m}}{b\mu_{m}}\\ 0 & 0 & 0 & 0 & 0\\ 0 & 0 & 0 & 0 & \frac{\beta_{f}r_{u}\Lambda_{f}}{b\mu_{f}}\\ 0 & 0 & 0 & 0 & 0\\ \beta_{c} & \beta_{c} & \beta_{c} & \beta_{c} & 0 \end{array}),\;V=(\begin{array}{ccccc} p_{m_{1}} & 0 & 0 & 0 & 0\\ -\alpha_{m}\theta_{m} & p_{m_{2}} & 0 & 0 & 0\\ 0 & 0 & p_{f_{1}} & 0 & 0\\ 0 & 0 & -\alpha_{f}\theta_{f} & p_{f_{2}} & 0\\ 0 & 0 & 0 & 0 & p_{w} \end{array}).$$ (5)

From (5), we compute the spectral radius of *FV*^−1^; hence


$$R_{0}\;=\;\rho (FV^{-1})\;=\;\sqrt{\;R_{c}\;(R_{m}+R_{f})\;},$$

$$\begin{array}{cc} {ℛ}_{c} & =\frac{\beta_{c}r_{u}}{bp_{w}},\;{ℛ}_{m}=\frac{\Lambda_{m}\beta_{m}(\alpha_{m}\theta_{m}+p_{m_{2}})}{\mu_{m}p_{m_{1}}p_{m_{2}}}\;\text{and}\;{ℛ}_{f}=\frac{\Lambda_{f}\beta_{f}(\alpha_{f}\theta_{f}+p_{f_{2}})}{\mu_{f}p_{f_{1}}p_{f_{2}}}. \end{array}$$ (6)

where ${ℛ}_{c}$ denotes the environmental influence, ${ℛ}_{m}$ being the influence by the males and ${ℛ}_{f}$ the influence by the females. Here, $p_{m_{1}}=\theta_{m}+\mu_{m}$, $p_{m_{2}}=\mu_{m}+v_{m}$, $p_{f_{1}}=\theta_{f}+\mu_{f}$, $p_{f_{2}}=\mu_{f}+v_{f}$ and $p_{w}=\phi +r_{c}$,

#### Epidemiological interpretation of term dominance.

Given our reproduction number $R_{0}=\sqrt{R_{c}\;(R_{m}+R_{f})}$, we presesent some scenario analysis in the absence of the numerical simulations:

- $R_{c}\gg R_{m},R_{f}$: transmission is environment-driven (contaminated-needle pathway dominates). Having some interventions that reduce contamination or increase clean-needle availability (e.g., sterilization, safe disposal of infected needles, expanding clean-needle supply) might have the largest effects.
- $R_{m}\gg R_{f}$: transmission is male-driven. Targeted reduction methods for men (reducing risky injections, lowering contact with contaminated needles, rapid testing/treatment for male PWID) will be more beneficial reducing spread.
- $R_{f}\gg R_{m}$: transmission is female-driven. Focused interventions for women (behavioral risk reduction, access to clean equipment, prompt diagnosis/treatment) are most impactful.
- Comparable $R_{m}\approx R_{f}$ with large *R*_*c*_: both sexes contribute but the environmental route has the largest effect, hence combined gender-sensitive methods plus needle-hygiene measures is indicated.
- Comparable $R_{m}\approx R_{f}$ with small *R*_*c*_: spread is mostly host-behavioral; balancing sex-specific risk-reduction and treatment coverage yields the largest benefit.

Using Theorem 2 in Diekmann and Heesterbeek [44], the following result is established.

**Theorem 3.2.** *The disease free equilibrium* ${ℰ}^{0}$ *of system (*1) *is locally asymptotically stable in* ${ℛ}_{0}<1$, *and unstable otherwise.*

In the following, we will discuss the global stability of the infection-free equilibrium ${ℰ}^{0}$.

**Theorem 3.3.** *If* ${ℛ}_{0}<1$, *the infection-free equilibrium is globally asymptotically stable in* Ω. *If* ${ℛ}_{0}>1$, *the system is uniformly persistent.*

*Proof*: Let $𝒴(t)=(A_{m},C_{m},A_{f},C_{f},W_{c})$. Since

$$\frac{dA_{m}}{dt}=\lambda_{m}S_{m}-(\mu_{m}+\theta_{m})A_{m},\;\frac{dC_{m}}{dt}=\alpha_{m}\theta_{m}A_{m}-(\mu_{m}+v_{m})C_{m},\;\frac{dA_{f}}{dt}=\lambda_{f}S_{f}-(\mu_{f}+\theta_{f})A_{f},\;\frac{dC_{f}}{dt}=\alpha_{f}\theta_{f}A_{f}-(\mu_{f}+v_{f})C_{f},\;\frac{dW_{c}}{dt}=\lambda_{c}W_{u}-(\phi +r_{c})W_{c},\;$$ (7)

it follows that


$$\dot{𝒴}=(F-V)𝒴$$

where *F* and *V* are defined in (5). It is worth noting that *F* and *V*^−1^ are non-negative. By the Perron-Frobenius Theorem ([45]), the non-negative matrix *V*^−1^*F* has a non-negative left eigenvector w≥0 with respect to $\rho (V^{-1}F)=\rho (FV^{-1})={ℛ}_{0},$ that is $w^{T}V^{-1}F={ℛ}_{0}w^{T}$. Motivated by Shuai et al. [46] , we define a Lyapunov function as follows


$$ℒ=w^{T}V^{-1}𝒴.$$

Differentiating ℒ along solutions of (3), we have

$$\dot{ℒ}=w^{T}V^{-1}\dot{𝒴}\leq w^{T}V^{-1}(F-V)𝒴=({ℛ}_{0}-1)w^{T}𝒴\leq 0\text{ if }{ℛ}_{0}\leq 1.$$ (8)

It can be easily verified that the largest invariant subset of Ω where $\dot{ℒ}=0$ is the singleton ${ℰ}^{0}$. Therefore, by LaSalle’s invariance principle [47], ${ℰ}^{0}$ is globally asymptotically stable in Ω when ${ℛ}_{0}\leq 1$. If ${ℛ}_{0}>1$, then by continuity, $\dot{ℒ}>0$ in a neighborhood of ${ℰ}^{0}$ in the interior of Ω. Solutions in the interior of Ω sufficiently close to ${ℰ}^{0}$ move away from the infection-free equilibrium, implying that the infection-free equilibrium is unstable. □

The result in Theorem 4 shows that ${ℛ}_{0}=1$ is a sharp threshold for disease dynamics: the disease will die out when ${ℛ}_{0}\leq 1$, whereas the disease will persist when ${ℛ}_{0}>1$. (We refer to [48–50] for more details on persistence theory.) Next, we turn to the analysis of the endemic equilibrium.

### 3.3 The endemic equilibrium

We now establish the endemic equilibrium of system (1). This is done by solving the following system (9) for $S_{i}^{*},\;A_{i}^{*},\;C_{i}^{*},\;R_{i}^{*},\;W_{u}^{*}\;,W_{c}^{*},\;i=m,f$ to obtain the endemic equilibrium point ${ℰ}^{*}=\left(S_{i}^{*},\;A_{i}^{*},\;C_{i}^{*},\;R_{i}^{*},\;W_{u}^{*}\;,W_{c}^{*}\right)$.

$$0=\Lambda_{i}-(\mu_{i}+\lambda_{i}^{*})S_{i}^{*},\;0=\lambda_{i}^{*}S_{i}-(\mu_{i}+\theta_{i})A_{i}^{*},\;0=\alpha_{i}\theta_{i}A_{i}^{*}-(\mu_{i}+v_{i})C_{i}^{*},\;0=(1-\alpha_{i})\theta_{i}A_{i}^{*}-\mu_{i}R_{i}^{*},\;0=b-(\lambda_{c}^{*}+r_{u})W_{u}^{*}+\phi W_{c}^{*},\;0=\lambda_{c}^{*}W_{u}^{*}-(\phi +r_{c})W_{c}^{*},\;i=m,f\;$$ (9)

with

$$\lambda_{i}^{*}=\frac{\beta_{i}W_{c}^{*}}{W_{u}^{*}+W_{c}^{*}},\;i=m,f$$ (10)

and

$$\lambda_{c}^{*}=\frac{\beta_{c}\left(A_{m}^{*}+A_{f}^{*}+C_{m}^{*}+C_{f}^{*}\right)}{W_{u}^{*}+W_{c}^{*}}.$$ (11)

From the third and fourth equations of (9) we have

$$C_{m}^{*}=\frac{\alpha_{m}\theta_{m}A_{m}^{*}}{p_{m_{2}}}\;\text{and}\;R_{m}^{*}=\frac{\theta_{m}\left(1-\alpha_{m}\right)A_{m}^{*}}{\mu_{m}}.$$ (12)

Combining the first and second equation of (9) and also combining the ninth and tenth equation of (9) we have

$$S_{m}^{*}=\frac{\Lambda_{m}-p_{m_{1}}A_{m}^{*}}{\mu_{m}}\;\text{and}\;W_{u}^{*}=\frac{b-r_{c}W_{c}^{*}}{r_{u}}.$$ (13)

Making use of the $W_{u}^{*}$ expression given in (%(13) and solving the second equation of (9) for $W_{c}^{*}$ in terms of $A_{m}^{*}$ gives

$$W_{c}^{*}=\frac{b\mu_{m}p_{m_{1}}A_{m}^{*}}{\beta_{m}\Lambda_{m}r_{u}-A_{m}^{*}p_{m_{1}}\left(r_{u}\left(\beta_{m}+\mu_{m}\right)-r_{c}\mu_{m}\right)}\Rightarrow W_{u}^{*}=\frac{b\left(p_{m_{1}}\left(\beta_{m}+\mu_{m}\right)A_{m}^{*}-\beta_{m}\Lambda_{m}\right)}{p_{m_{1}}\left(r_{u}\left(\beta_{m}+\mu_{m}\right)-r_{c}\mu_{m}\right)A_{m}^{*}-\beta_{m}\Lambda_{m}r_{u}}.$$ (14)

From the seventh and eighth equations of (9) we have

$$C_{f}^{*}=\frac{\alpha_{f}\theta_{f}A_{f}^{*}}{p_{f_{2}}}\;\text{and}\;R_{f}^{*}=\frac{\left(1-\alpha_{f}\right)\theta_{f}A_{f}^{*}}{\mu_{f}}.$$ (15)

From the fifth and sixth equation of (9) we have

$$\begin{array}{cc} & S_{f}^{*}=\frac{\Lambda_{f}-A_{f}^{*}p_{f_{1}}}{\mu_{f}}\;\text{and}\;A_{f}^{*}=\frac{\beta_{f}\Lambda_{f}\mu_{m}p_{m_{1}}A_{m}^{*}}{p_{f_{1}}\left(A_{m}p_{m_{1}}\left(\beta_{f}\mu_{m}-\mu_{f}\beta_{m}\right)+\mu_{f}\beta_{m}\Lambda_{m}\right)},\\ \Rightarrow & S_{f}^{*}=\frac{\Lambda_{f}\beta_{m}\left(\Lambda_{m}-p_{m_{1}}A_{m}^{*}\right)}{A_{m}p_{m_{1}}\left(\beta_{f}\mu_{m}-\mu_{f}\beta_{m}\right)+\mu_{f}\beta_{m}\Lambda_{m}}. \end{array}$$ (16)

Using the ninth equation of (9), we obtain the following fourth order polynomial equation in terms of $A_{m}^{*}$:

$$A_{m}^{*}\left(\xi_{3}A_{m}^{*3}+\xi_{2}A_{m}^{*2}+\xi_{1}A_{m}^{*}+\xi_{0}\right)=0.$$ (17)

Solving (17) gives $A_{m}^{*}=0$ which corresponds to the disease-free equilibrium, or

$$\begin{array}{cc} & \xi_{3}A_{m}^{*3}+\xi_{2}A_{m}^{*2}+\xi_{1}A_{m}^{*}+\xi_{0}=0\;\text{where}\\ \xi_{0} & =bp_{m_{1}}p_{m_{2}}p_{f_{1}}p_{f_{2}}p_{w}\mu_{f}\mu_{m}\left({ℛ}_{0}+1\right)\left({ℛ}_{0}-1\right),\\ \xi_{1} & =b\beta_{m}\Lambda_{m}p_{m_{1}}(p_{f_{1}}p_{f_{2}}(\beta_{m}\mu_{m}(\mu_{f}(2bp_{m_{1}}p_{m_{2}}p_{w}+\beta_{c}\Lambda_{m}(r_{c}-2r_{u})(\alpha_{m}\theta_{m}+p_{m_{2}}))\\ & +\beta_{c}\beta_{f}\Lambda_{m}r_{u}(\alpha_{m}\theta_{m}+p_{m_{2}}))-b\beta_{f}\mu_{m}^{2}p_{m_{1}}p_{m_{2}}p_{w}-3\beta_{c}\mu_{f}\beta_{m}^{2}\Lambda_{m}r_{u}(\alpha_{m}\theta_{m}+p_{m_{2}}))\\ & -\beta_{c}\beta_{f}\Lambda_{f}\mu_{m}p_{m_{1}}p_{m_{2}}(\alpha_{f}\theta_{f}+p_{f_{2}})(2r_{u}(\beta_{m}+\mu_{m})-r_{c}\mu_{m})),\\ \xi_{2} & =bp_{m_{1}}^{2}(p_{f_{1}}p_{f_{2}}\beta_{m}(p_{m_{2}}(\mu_{m}(bp_{m_{1}}p_{w}(\beta_{f}\mu_{m}-\mu_{f}\beta_{m})+\beta_{c}r_{c}\Lambda_{m}(\beta_{f}\mu_{m}-\mu_{f}(2\beta_{m}+\mu_{m})))+\beta_{c}\Lambda_{m}r_{u}\times \\ & (\beta_{m}+\mu_{m})(3\mu_{f}\beta_{m}+\mu_{m}(\mu_{f}-2\beta_{f})))+\beta_{c}\alpha_{m}\theta_{m}\Lambda_{m}(r_{c}\mu_{m}(\beta_{f}\mu_{m}-\mu_{f}(2\beta_{m}+\mu_{m}))+r_{u}(\beta_{m}+\mu_{m})\times \\ & (3\mu_{f}\beta_{m}+\mu_{m}(\mu_{f}-2\beta_{f}))))+\beta_{c}\beta_{f}\Lambda_{f}\mu_{m}p_{m_{1}}p_{m_{2}}(\beta_{m}+\mu_{m})(\alpha_{f}\theta_{f}+p_{f_{2}})(r_{u}(\beta_{m}+\mu_{m})-r_{c}\mu_{m})),\\ \xi_{3} & =-b\beta_{c}p_{f_{1}}p_{f_{2}}p_{m_{1}}^{3}(\beta_{m}+\mu_{m})(\mu_{f}\beta_{m}-\beta_{f}\mu_{m})(\alpha_{m}\theta_{m}+p_{m_{2}})(r_{u}(\beta_{m}+\mu_{m})-r_{c}\mu_{m}). \end{array}$$ (18)

Take note that if ${ℛ}_{0}>1$ then $\xi_{0}>0$, while if ${ℛ}_{0}<1$ then $\xi_{0}<0$. Using Descartes’ rule of signs, we have the following possibilities (Table 2) on the number of roots of polynomial equation in (18).

**Table 2:** Number of positive roots.

	$\xi_{3}^{+}$								$\xi_{3}^{-}$							
	$\xi_{2}^{+}$				$\xi_{2}^{-}$				$\xi_{2}^{+}$				$\xi_{2}^{-}$			
	$\xi_{1}^{+}$		$\xi_{1}^{-}$		$\xi_{1}^{+}$		$\xi_{1}^{-}$		$\xi_{1}^{+}$		$\xi_{1}^{-}$		$\xi_{1}^{+}$		$\xi_{1}^{-}$	
	$\xi_{0}^{+}$	$\xi_{0}^{-}$	$\xi_{0}^{+}$	$\xi_{0}^{-}$	$\xi_{0}^{+}$	$\xi_{0}^{-}$	$\xi_{0}^{+}$	$\xi_{0}^{-}$	$\xi_{0}^{+}$	$\xi_{0}^{-}$	$\xi_{0}^{+}$	$\xi_{0}^{-}$	$\xi_{0}^{+}$	$\xi_{0}^{-}$	$\xi_{0}^{+}$	$\xi_{0}^{-}$
	$\left({ℛ}_{0}>1\right)$	$\left({ℛ}_{0}<1\right)$	$\left({ℛ}_{0}>1\right)$	$\left({ℛ}_{0}<1\right)$	$\left({ℛ}_{0}>1\right)$	$\left({ℛ}_{0}<1\right)$	$\left({ℛ}_{0}>1\right)$	$\left({ℛ}_{0}<1\right)$	$\left({ℛ}_{0}>1\right)$	$\left({ℛ}_{0}<1\right)$	$\left({ℛ}_{0}>1\right)$	$\left({ℛ}_{0}<1\right)$	$\left({ℛ}_{0}>1\right)$	$\left({ℛ}_{0}<1\right)$	$\left({ℛ}_{0}>1\right)$	$\left({ℛ}_{0}<1\right)$
***i*** ^*^	**0**	**1**	**2**	**1**	**2**	**3**	**2**	**1**	**1**	**2**	**3**	**2**	**1**	**2**	**1**	**0**

Here i^*^ denotes the number of possible roots with, $\xi_{i}^{+}\Leftrightarrow \xi_{i}>0$ for i=0,1,2,3 and $\xi_{i}^{-}\Leftrightarrow \xi_{i}<0$ for i=0,1,2,3.

#### 3.3.1 Local stability of the endemic equilibrium.

We now establish the local asymptotic stability of the endemic equilibrium point ${ℰ}^{*}$ of system (1). Conditions for establishing local stability of ${ℰ}^{*}$ are obtained through application of the centre manifold theorem (Theorem 4.1) proven in Castillo-Chavez and Song [51]. We avoid rewriting the elaborate form of the theorem and refer readers to [51] for more details about this theorem.

We introduce the following new variable notations:

$S_{m}=x_{1},\;A_{m}=x_{2},\;C_{m}=x_{3},\;R_{m}=x_{4},\;S_{f}=x_{5},\;A_{f}=x_{6},\;C_{f}=x_{7},\;R_{f}=x_{8},\;W_{u}=x_{9},\;W_{c}=x_{10}$. We conveniently use the vector notation $X=(x_{1},x_{2},x_{3},x_{4},x_{5},x_{6},x_{7},x_{8},x_{9},x_{10}{)}^{T}$. Thus, system (1) takes the form $\frac{dX}{dt}=F(t,x(t))=(f_{1},f_{2},f_{3},f_{4},f_{5},f_{6},f_{7},f_{8},f_{9},f_{10}{)}^{T}$, where

$$\left\{ \begin{array}{cc} \frac{dx_{i}}{dt} & =\Lambda_{j}-(\mu_{j}+\lambda_{j})x_{i}=f_{i},\;\{i=1,j=m\}\;\text{and}\;\{i=5,j=f\}\\ \frac{dx_{i}}{dt} & =\lambda_{j}x_{i-1}-(\mu_{j}+\theta_{j})x_{i}=f_{i},\;\{i=2,j=m\}\;\text{and}\;\{i=6,j=f\}\\ \frac{dx_{i}}{dt} & =\alpha_{j}\theta_{j}x_{i-1}-(\mu_{j}+v_{j})x_{i}=f_{i},\;\{i=3,j=m\}\;\text{and}\;\{i=7,j=f\}\\ \frac{dx_{i}}{dt} & =(1-\alpha_{j})\theta_{j}x_{i-2}-\mu_{j}x_{i}=f_{i},\;\{i=4,j=m\}\;\text{and}\;\{i=8,j=f\}\\ \frac{dx_{9}}{dt} & =b-(\lambda_{c}+r_{u})x_{9}+\phi x_{10}=f_{9},\\ \frac{dx_{10}}{dt} & =\lambda_{c}x_{9}-(\phi +r_{c})x_{10}=f_{10}, \end{array}\right.$$ (19)

where

$$\lambda_{j}=\frac{\beta_{j}x_{10}}{x_{9}+x_{10}},\;j=m,f\;\text{and}\;\lambda_{c}=\frac{\beta_{c}(x_{2}+x_{6}+x_{3}+x_{7})}{x_{9}+x_{10}}.$$ (20)

We now define

$$\beta_{j}=\varepsilon_{j}\beta_{c},\;j=m,f$$ (21)

with $\varepsilon_{j}=1\Rightarrow \beta_{c}=\beta_{j}$,    $\varepsilon_{j}\in (0,1)\Rightarrow \beta_{j}>\beta_{c}$   and   $\varepsilon_{j}>1\Rightarrow \beta_{j}<\beta_{c}$.

Let $\beta_{c}$ be the bifurcation parameter, ${ℛ}_{0}=1$ corresponds to

$$\beta_{c}=\beta_{c}^{*}=\sqrt{\frac{bp_{w}}{r_{u}\left(\frac{\Lambda_{f}\epsilon_{f}\left(\alpha_{f}\theta_{f}+p_{f_{2}}\right)}{\mu_{f}p_{f_{1}}p_{f_{2}}}+\frac{\Lambda_{m}\epsilon_{m}\left(\alpha_{m}\theta_{m}+p_{m_{2}}\right)}{\mu_{m}p_{m_{1}}p_{m_{2}}}\right)}}.$$ (22)

The Jacobian matrix of system (19) at ${ℰ}^{0}$ when $\beta_{c}=\beta_{c}^{*}$ is given by


$$J^{*}({ℰ}^{0})=\left(\begin{array}{cccccccccc} -\mu_{m} & 0 & 0 & 0 & 0 & 0 & 0 & 0 & 0 & -\frac{\beta_{c}^{*}\Lambda_{m}\epsilon_{m}r_{u}}{b\mu_{m}}\\ 0 & -p_{m_{1}} & 0 & 0 & 0 & 0 & 0 & 0 & 0 & \frac{\beta_{c}^{*}\Lambda_{m}\epsilon_{m}r_{u}}{b\mu_{m}}\\ 0 & \alpha_{m}\theta_{m} & -p_{m_{2}} & 0 & 0 & 0 & 0 & 0 & 0 & 0\\ 0 & \left(1-\alpha_{m}\right)\theta_{m} & 0 & -\mu_{m} & 0 & 0 & 0 & 0 & 0 & 0\\ 0 & 0 & 0 & 0 & -\mu_{f} & 0 & 0 & 0 & 0 & -\frac{\beta_{c}^{*}\Lambda_{f}\epsilon_{f}r_{u}}{b\mu_{f}}\\ 0 & 0 & 0 & 0 & 0 & -p_{f_{1}} & 0 & 0 & 0 & \frac{\beta_{c}^{*}\Lambda_{f}\epsilon_{f}r_{u}}{b\mu_{f}}\\ 0 & 0 & 0 & 0 & 0 & \alpha_{f}\theta_{f} & -p_{f_{2}} & 0 & 0 & 0\\ 0 & 0 & 0 & 0 & 0 & \left(1-\alpha_{f}\right)\theta_{f} & 0 & -\mu_{f} & 0 & 0\\ 0 & -\beta_{c}^{*} & -\beta_{c}^{*} & 0 & 0 & -\beta_{c}^{*} & -\beta_{c}^{*} & 0 & -r_{u} & \phi \\ 0 & \beta_{c}^{*} & \beta_{c}^{*} & 0 & 0 & \beta_{c}^{*} & \beta_{c}^{*} & 0 & 0 & -p_{w} \end{array}\right)$$

where $p_{f_{1}},\;p_{f_{2}},\;p_{m_{1}},\;p_{m_{2}}$ and *p*_*w*_ are defined as before.

System (19), has a simple eigenvalue at $\beta_{c}=\beta_{c}^{*}$. Thus, we can apply the center manifold theory in order to analyse the dynamics of system (1) near $\beta_{c}=\beta_{c}^{*}$. The following are components of the right eigenvector $w=(w_{1},w_{2},w_{3},w_{4},w_{5},w_{6},w_{7},w_{8},w_{9},w_{10}{)}^{T}$ of $J^{*}({ℰ}^{0})$.


$$\begin{array}{ccc} w_{1} & =-\beta_{c}^{*}\mu_{f}^{2}p_{f_{1}}p_{f_{2}}\Lambda_{m}p_{m_{1}}p_{m_{2}}\epsilon_{m}r_{u}^{2},\; & w_{2}=\beta_{c}^{*}\mu_{f}^{2}p_{f_{1}}p_{f_{2}}\Lambda_{m}\mu_{m}p_{m_{2}}\epsilon_{m}r_{u}^{2},\\ w_{3} & =\beta_{c}^{*}\mu_{f}^{2}p_{f_{1}}p_{f_{2}}\alpha_{m}\theta_{m}\Lambda_{m}\mu_{m}\epsilon_{m}r_{u}^{2},\; & w_{4}=\beta_{c}^{*}\mu_{f}^{2}p_{f_{1}}p_{f_{2}}\left(1-\alpha_{m}\right)\theta_{m}\Lambda_{m}p_{m_{2}}\epsilon_{m}r_{u}^{2},\\ w_{5} & =-\beta_{c}^{*}\Lambda_{f}\epsilon_{f}p_{f_{1}}p_{f_{2}}\mu_{m}^{2}p_{m_{1}}p_{m_{2}}r_{u}^{2},\; & w_{6}=\beta_{c}^{*}\Lambda_{f}\mu_{f}\epsilon_{f}p_{f_{2}}\mu_{m}^{2}p_{m_{1}}p_{m_{2}}r_{u}^{2},\\ w_{7} & =\beta_{c}^{*}\alpha_{f}\theta_{f}\Lambda_{f}\mu_{f}\epsilon_{f}\mu_{m}^{2}p_{m_{1}}p_{m_{2}}r_{u}^{2},\; & w_{8}=\beta_{c}^{*}\left(1-\alpha_{f}\right)\theta_{f}\Lambda_{f}\epsilon_{f}p_{f_{2}}\mu_{m}^{2}p_{m_{1}}p_{m_{2}}r_{u}^{2},\\ w_{9} & =-b\mu_{f}^{2}p_{f_{1}}p_{f_{2}}\mu_{m}^{2}p_{m_{1}}p_{m_{2}}r_{c},\; & w_{10}=b\mu_{f}^{2}p_{f_{1}}p_{f_{2}}\mu_{m}^{2}p_{m_{1}}p_{m_{2}}r_{u}. \end{array}$$

The following are components of the left eigenvector $v=(v_{1},v_{2},v_{3},v_{4},v_{5},v_{6},v_{7},v_{8},v_{9},v_{10}{)}^{T}$ of $J^{*}({ℰ}^{0})$, associated with the zero eigenvalue at $\beta_{c}=\beta_{c}^{*}$.


$$v_{2}=\beta_{c}^{*}p_{f_{1}}p_{f_{2}}\left(\alpha_{m}\theta_{m}+p_{m_{2}}\right),\;v_{3}=\beta_{c}^{*}p_{f_{1}}p_{f_{2}}p_{m_{1}},\;v_{6}=\beta_{c}^{*}p_{m_{1}}p_{m_{2}}\left(\alpha_{f}\theta_{f}+p_{f_{2}}\right),\;v_{7}=\beta_{c}^{*}p_{f_{1}}p_{m_{1}}p_{m_{2}},$$

$$v_{10}=p_{f_{1}}p_{f_{2}}p_{m_{1}}p_{m_{2}},\;v_{1}=v_{4}=v_{5}=v_{8}=v_{9}=0.$$

We now compute the terms **a** and **b** and apply Theorem 4.1 in Castillo-Chavez and Song (2004). The following are the associated non-zero partial derivatives of *F* for system (19) at the disease-free equilibrium ${ℰ}^{0}$.

$$\begin{array}{cc} \frac{\partial^{2}f_{1}}{\partial x_{1}\partial x_{10}} & =\frac{\partial^{2}f_{1}}{\partial x_{10}\partial x_{1}}=-\frac{\beta_{c}^{*}\epsilon_{m}r_{u}}{b},\;\frac{\partial^{2}f_{1}}{\partial x_{9}\partial x_{10}}=\frac{\partial^{2}f_{1}}{\partial x_{10}\partial x_{9}}=\frac{\beta_{c}^{*}\Lambda_{m}\epsilon_{m}r_{u}^{2}}{b^{2}\mu_{m}},\;\frac{\partial^{2}f_{1}}{\partial x_{10}^{2}}=\frac{2\beta_{c}^{*}\Lambda_{m}\epsilon_{m}r_{u}^{2}}{b^{2}\mu_{m}},\\ \frac{\partial^{2}f_{2}}{\partial x_{1}\partial x_{10}} & =\frac{\partial^{2}f_{2}}{\partial x_{10}\partial x_{1}}=\frac{\beta_{c}^{*}\epsilon_{m}r_{u}}{b},\;\frac{\partial^{2}f_{2}}{\partial x_{9}\partial x_{10}}=\frac{\partial^{2}f_{2}}{\partial x_{10}\partial x_{9}}=-\frac{\beta_{c}^{*}\Lambda_{m}\epsilon_{m}r_{u}^{2}}{b^{2}\mu_{m}},\;\frac{\partial^{2}f_{2}}{\partial x_{10}^{2}}=-\frac{2\beta_{c}^{*}\Lambda_{m}\epsilon_{m}r_{u}^{2}}{b^{2}\mu_{m}},\\ \frac{\partial^{2}f_{5}}{\partial x_{5}\partial x_{10}} & =\frac{\partial^{2}f_{5}}{\partial x_{10}\partial x_{5}}=-\frac{\beta_{c}^{*}\epsilon_{f}r_{u}}{b},\;\frac{\partial^{2}f_{5}}{\partial x_{9}\partial x_{10}}=\frac{\partial^{2}f_{5}}{\partial x_{10}\partial x_{9}}=\frac{\beta_{c}^{*}\Lambda_{f}\epsilon_{f}r_{u}^{2}}{b^{2}\mu_{f}},\;\frac{\partial^{2}f_{5}}{\partial x_{10}^{2}}=\frac{2\beta_{c}^{*}\Lambda_{f}\epsilon_{f}r_{u}^{2}}{b^{2}\mu_{f}},\\ \frac{\partial^{2}f_{6}}{\partial x_{5}\partial x_{10}} & =\frac{\partial^{2}f_{6}}{\partial x_{10}\partial x_{5}}=\frac{\beta_{c}^{*}\epsilon_{f}r_{u}}{b},\;\frac{\partial^{2}f_{6}}{\partial x_{9}\partial x_{10}}=\frac{\partial^{2}f_{6}}{\partial x_{10}\partial x_{9}}=-\frac{\beta_{c}^{*}\Lambda_{f}\epsilon_{f}r_{u}^{2}}{b^{2}\mu_{f}},\;\frac{\partial^{2}f_{6}}{\partial x_{10}^{2}}=-\frac{2\beta_{c}^{*}\Lambda_{f}\epsilon_{f}r_{u}^{2}}{b^{2}\mu_{f}},\\ \frac{\partial^{2}f_{9}}{\partial x_{2}\partial x_{10}} & =\frac{\partial^{2}f_{9}}{\partial x_{10}\partial x_{2}}=\frac{\beta_{c}^{*}r_{u}}{b},\;\frac{\partial^{2}f_{9}}{\partial x_{3}\partial x_{10}}=\frac{\partial^{2}f_{9}}{\partial x_{10}\partial x_{3}}=\frac{\beta_{c}^{*}r_{u}}{b},\;\frac{\partial^{2}f_{9}}{\partial x_{6}\partial x_{10}}=\frac{\partial^{2}f_{9}}{\partial x_{10}\partial x_{6}}=\frac{\beta_{c}^{*}r_{u}}{b},\\ \frac{\partial^{2}f_{9}}{\partial x_{7}\partial x_{10}} & =\frac{\partial^{2}f_{9}}{\partial x_{10}\partial x_{7}}=\frac{\beta_{c}^{*}r_{u}}{b},\;\frac{\partial^{2}f_{10}}{\partial x_{2}\partial x_{10}}=\frac{\partial^{2}f_{10}}{\partial x_{10}\partial x_{2}}=-\frac{\beta_{c}^{*}r_{u}}{b},\;\frac{\partial^{2}f_{10}}{\partial x_{3}\partial x_{10}}=\frac{\partial^{2}f_{10}}{\partial x_{10}\partial x_{3}}=-\frac{\beta_{c}^{*}r_{u}}{b},\\ \frac{\partial^{2}f_{10}}{\partial x_{6}\partial x_{10}} & =\frac{\partial^{2}f_{10}}{\partial x_{10}\partial x_{6}}=-\frac{\beta_{c}^{*}r_{u}}{b},\;\frac{\partial^{2}f_{10}}{\partial x_{7}\partial x_{10}}=\frac{\partial^{2}f_{10}}{\partial x_{10}\partial x_{7}}=-\frac{\beta_{c}^{*}r_{u}}{b},\;\frac{\partial^{2}f_{1}}{\partial x_{10}\partial \beta_{c}^{*}}=-\frac{\Lambda_{m}\epsilon_{m}r_{u}}{b\mu_{m}},\\ \frac{\partial^{2}f_{2}}{\partial x_{10}\partial \beta_{c}^{*}} & =\frac{\Lambda_{m}\epsilon_{m}r_{u}}{b\mu_{m}},\;\frac{\partial^{2}f_{5}}{\partial x_{10}\partial \beta_{c}^{*}}=-\frac{\Lambda_{f}\epsilon_{f}r_{u}}{b\mu_{f}},\;\frac{\partial^{2}f_{6}}{\partial x_{10}\partial \beta_{c}^{*}}=\frac{\Lambda_{f}\epsilon_{f}r_{u}}{b\mu_{f}},\\ \frac{\partial^{2}f_{9}}{\partial x_{2}\partial \beta_{c}^{*}} & =\frac{\partial^{2}f_{9}}{\partial x_{3}\partial \beta_{c}^{*}}=\frac{\partial^{2}f_{9}}{\partial x_{6}\partial \beta_{c}^{*}}=\frac{\partial^{2}f_{9}}{\partial x_{7}\partial \beta_{c}^{*}}=-1,\;\frac{\partial^{2}f_{10}}{\partial x_{2}\partial \beta_{c}^{*}}=\frac{\partial^{2}f_{10}}{\partial x_{3}\partial \beta_{c}^{*}}=\frac{\partial^{2}f_{10}}{\partial x_{6}\partial \beta_{c}^{*}}=\frac{\partial^{2}f_{10}}{\partial x_{7}\partial \beta_{c}^{*}}=1, \end{array}$$ (23)

It thus follows that


$$\text{a}=2v_{2}w_{1}w_{10}\frac{\partial^{2}f_{2}}{\partial x_{1}\;\partial x_{10}}+2v_{2}w_{9}w_{10}\frac{\partial^{2}f_{2}}{\partial x_{9}\;\partial x_{10}}+v_{2}w_{10}w_{10}\frac{\partial^{2}f_{2}}{\partial x_{10}\;\partial x_{10}}+2v_{6}w_{5}w_{10}\frac{\partial^{2}f_{6}}{\partial x_{5}\;\partial x_{10}}$$

$$+2v_{6}w_{9}w_{10}\frac{\partial^{2}f_{6}}{\partial x_{9}\;\partial x_{10}}+v_{6}w_{10}w_{10}\frac{\partial^{2}f_{6}}{\partial x_{10}\;\partial x_{10}}+2v_{10}w_{2}w_{10}\frac{\partial^{2}f_{10}}{\partial x_{2}\;\partial x_{10}}+2v_{10}w_{3}w_{10}\frac{\partial^{2}f_{10}}{\partial x_{3}\;\partial x_{10}}$$

$$+2v_{10}w_{6}w_{10}\frac{\partial^{2}f_{10}}{\partial x_{6}\;\partial x_{10}}+2v_{10}w_{7}w_{10}\frac{\partial^{2}f_{10}}{\partial x_{7}\;\partial x_{10}}$$

$$=-\frac{1}{X}2\beta_{c}\mu_{f}^{3}p_{f_{1}}^{2}p_{f_{2}}^{2}\mu_{m}^{3}p_{m_{1}}^{2}p_{m_{2}}^{2}r_{u}^{3}(\mu_{f}p_{f_{1}}p_{f_{2}}\Lambda_{m}\epsilon_{m}\left(\alpha_{m}\theta_{m}+p_{m_{2}}\right)\left(b\mu_{f}p_{f_{1}}p_{f_{2}}p_{m_{1}}p_{m_{2}}\epsilon_{m}p_{w}+XY\left(2r_{u}-r_{c}\right)\right)$$

$$+b\Lambda_{f}\epsilon_{f}^{2}p_{f_{1}}p_{f_{2}}\mu_{m}^{2}p_{m_{1}}^{2}p_{m_{2}}^{2}p_{w}\left(\alpha_{f}\theta_{f}+p_{f_{2}}\right)+XY\Lambda_{f}\epsilon_{f}\mu_{m}p_{m_{1}}p_{m_{2}}\left(2r_{u}-r_{c}\right)\left(\alpha_{f}\theta_{f}+p_{f_{2}}\right)),$$

where


$$\begin{array}{c} X=\mu_{f}p_{f_{1}}p_{f_{2}}\Lambda_{m}\epsilon_{m}\left(\alpha_{m}\theta_{m}+p_{m_{2}}\right)+\Lambda_{f}\epsilon_{f}\mu_{m}p_{m_{1}}p_{m_{2}}\left(\alpha_{f}\theta_{f}+p_{f_{2}}\right)\;\text{and}\;Y=\sqrt{\frac{b\mu_{f}p_{f_{1}}p_{f_{2}}\mu_{m}p_{m_{1}}p_{m_{2}}p_{w}}{Xr_{u}}}. \end{array}$$

Note that if $r_{u}>r_{c}$, then a<0 and a}>0 if $r_{c}>r_{u}$. Lastly,


$$\text{b}=\frac{2\sqrt{b^{2}X\mu_{f}^{4}p_{f_{1}}^{4}p_{f_{2}}^{4}\mu_{m}^{4}p_{m_{1}}^{4}p_{m_{2}}^{4}p_{w}^{2}r_{u}^{3}}}{\sqrt{b\mu_{f}p_{f_{1}}p_{f_{2}}\mu_{m}p_{m_{1}}p_{m_{2}}p_{w}}}>0.$$

We thus have the following result

**Theorem 3.4.** *If* $r_{u}>r_{c}$, *then the endemic equilibrium* ${ℰ}^{*}$ *is locally asymptotically stable for* ${ℛ}_{0}>1$ *but close to one. Otherwise, if* $r_{c}>r_{u}$ *then system* (1) *has a backward bifurcation at* ${ℛ}_{0}=1$.

**Remark:** When the model exhibits backward bifurcation, reducing ${ℛ}_{0}$ below unit is not sufficient to control the HCV epidemic. Backward bifurcation happens when the model’s dynamics allow the disease to persist even if ${ℛ}_{0}<1$, due to the presence of multiple stable states (endemic equilibria) alongside the disease-free state. This can occur, for example, when the discard rate of contaminated needles (*r*_*c*_) exceeds that of uncontaminated needles (*r*_*u*_), or when other factors like the rate of needle contamination ($\beta_{c}$) or sex-specific contact rates ($\beta_{m}$, $\beta_{f}$) create complex interactions. In simple terms, even if the basic reproduction number drops below 1, the epidemic might not die out if initial infection levels or environmental conditions (e.g., needle reuse) are high. This means stronger, combined efforts, such as improving needle disposal, reducing risky behaviors, and stengthening recovery support are needed to push the system below a critical threshold to eliminate the disease.

### 3.4 Parameter estimation

In this section, we perform numerical simulations and sensitivity analyses on our model parameters. This modeling study relies entirely on secondary data extracted from peer-reviewed publications and official public health reports (e.g., WHO, CDC), all of which are cited in the bibliography. All sources are openly accessible online via provided DOIs, PubMed IDs, or institutional URLs. No new patient data were collected or accessed. Thus, no ethical clearance was necessary.

We begin by presenting our table of parameters, which have been mostly derived from the studies by Wang et al. [52] and Miller-Dickson et al. [53]. Some of the parameters were estimated based on data from the USA [54], and these values are highlighted in the Table below. The initial conditions employed in our analysis are outlined as follows: $S_{i}(0)=8500000,\;A_{m}=452,\;A_{f}=307,\;C_{i}=1,\;R_{i}(0)=0,\;W_{u}(0)=11000000,\;W_{c}=2000$, based on HCV data provided in Supplementary materials (Appendix C) for males and females in the USA.

**Fig 1:**
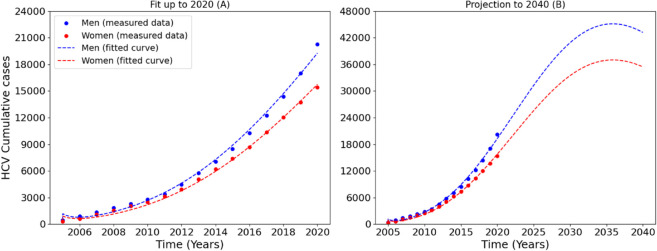
Data fitting results for the HCV dynamics model. Blue markers represent observed data points for men, and red markers represent observed data points for women. The blue line shows the model’s fitted curve for men, while the red line illustrates the model’s predictions for women. In Fig 1, panel A (left) displays the basic fitting based on available data, while panel B (right) extends the fitting to include projections up to the year 2040. The data used in this analysis are from the USA. Visual inspection of the fitted trajectories indicates that the model successfully reproduces the overall epidemic trend for both sexes, with RMS = 1025.28543 and NRMS = 0.21. Full computations for the RMS and NRMS are provided in the Supplementary materials (Appendix B).

Fig 1 shows the sex-stratified model fitting and long-term projections of cumulative HCV cases in the USA. Panel A shows the fittings of the HCV model vs observed cumulative case data for men (blue) and women (red) from 2005 to 2020. The model fits well the observed data. The steeper curve observed for men indicates a higher rate of HCV accumulation compared to women, consistent with known sex-specific risk factors. It has also been seen that the rate of current illicit drug use is higher for males than for females and males are more likely than females to be current users of several different illicit drugs [59]. Panel B extends the fitted model to project future trends up to 2040, and the projections indicate a continued increase in cumulative cases for both sexes until reaching peak levels around 2035, after then they begin to decline gradual. Importantly, the model predicts a persistent disparity, with men continuing to bear a disproportionately higher burden of HCV. The quality of fit and the projected trends highlight the utility of the model for long-term forecasting and suggest the need for sustained, sex-specific public health interventions. The divergence in trajectories beyond 2020 also highlights the potential consequences of inaction or unequal treatment outreach post 2020.

## 4 Results

In this section we present the results of our study, which are the numerical simulations based on the parameters presented in Table 3. A comprehensive summary of the model findings and their implications is provided in the Supplementary Material (Appendix D). Firstly, we present the sensitivity analysis, using the Partial Rank Correlation Coefficient (PRCC) analysis.

**Table 3:** Parameters used in the HCV dynamics model (units = years). The table lists both estimated parameters, derived from our data fitting procedures, and fixed parameters based on prior literature. These parameters form the basis for the simulations and projections made in our study. Model parameters were assumed constant over time to reflect average epidemiological conditions and ensure analytical tractability. However, key parameters were varied in simulations to capture plausible behavioural and intervention-driven changes in transmission dynamics.

Definition	Symbol	Value	Source
Recruitment rate	$\Lambda_{i}$	17155	[53]
Injection rate times infection of needle probability	$\beta_{c}$	16.240084	Fitted
Injection rate times infection of host rate (male)	$\beta_{m}$	0.98427	Fitted
Injection rate times infection of host rate (female)	$\beta_{f}$	0.761899	Fitted
Rate of removal	$\mu_{i}$	0.01825	[55]
Rate of acute becoming chronic	$\theta_{i}$	2	[56]
Proportion of those that recover	$\alpha_{i}$	varied (0–1)	Estimated
Recruitment rate of uninfected needles	*b*	1146100	[57,58]
Fractional decay rate of HCV infection in needles	*ϕ*	427	[42]
Discard rate of uninfected needles	*r*_*u*_	0.065776	Fitted
Discard rate of infected needles	*r*_*c*_	0.010003	Fitted

To assess the sensitivity of model outcomes to parameter uncertainty, we performed a PRCC analysis. This approach quantifies the strength and direction of monotonic relationships between input parameters and model output, while accounting for the influence of other variables. We generated 20000 samples of parameter sets using Latin Hypercube Sampling to ensure comprehensive coverage of the parameter space. The model was evaluated for each sample to compute the peak prevalence of acute infection. PRCC values were then calculated to identify the most influential parameters affecting the outcome. This analysis provides insight into which biological or behavioral factors most significantly drive model dynamics, guiding future data collection and intervention priorities.

**Fig 2:**
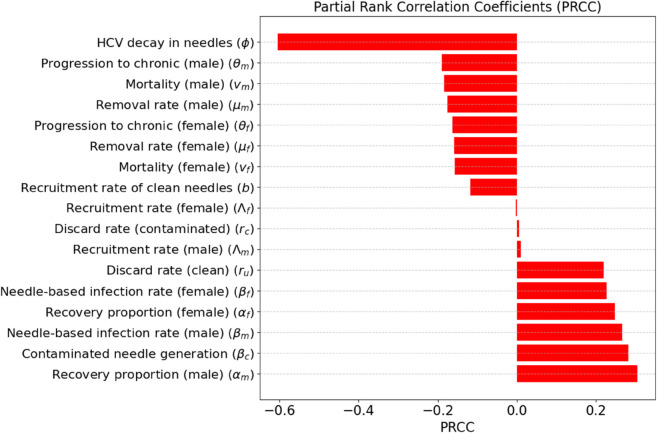
The PRCC analysis was performed using 20000 Latin Hypercube samples to evaluate the sensitivity of the model output, the peak prevalence of acute infection, to each input parameter. Parameters with higher absolute PRCC values have a stronger influence on the outcome. Specifically, positive values indicate a direct relationship with the output, while negative values indicate an inverse relationship.

The PRCC analysis (Fig 2) reveals that the most influential parameters affecting the peak prevalence of acute infection are the recovery proportion in males ($\alpha_{m}$), contaminated needle generation rate ($\beta_{c}$), and needle-based infection rates in males and females ($\beta_{m}$, $\beta_{f}$), all of which show strong positive correlations. This suggests that increases in these parameters are associated with higher levels of infections. Conversely, the HCV decay rate in needles (*ϕ*) and progression to chronic infection in males ($\theta_{m}$) exhibit strong negative correlations, indicating that faster needle decontamination and reduced progression rates may help suppress infection prevalence. These findings indicate the critical role of behavioral and environmental parameters particularly those related to needle dynamics and gender-specific recovery in shaping epidemic outcomes. For the health authorities, this shows the importance of gender targeted recovery interventions and harm reduction strategies, such as improving needle exchange programs and reducing contaminated needle circulation to effectively curtail HCV transmission.

**Fig 3:**
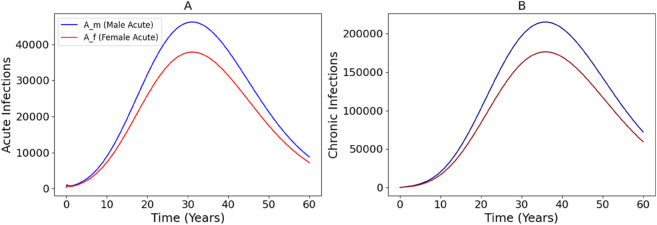
Trends of acute and chronic HCV infections over a 60-year period, stratified by sex. Panel A shows the number of acute infections for males (blue) and females (red); Panel B illustrates the corresponding chronic infections.

Fig 3 illustrates the dynamics of HCV infections stratified by sex, with acute infections shown on the left (Panel A) and chronic infections on the right (Panel B). Both male and female populations exhibit a rise in acute infections during the initial years, peaking around year 30 for females and 35-40 for males before gradually declining. Notably, males experience a higher burden of acute infections compared to females, likely due to differences in behavioral or biological susceptibility (needle sharing). This disparity carries over to chronic infections, where the male population also shows a higher peak, occurring slightly later around year 35-40 years. The time lag between the acute and chronic peaks reflects the natural progression of HCV from acute to chronic stages. These trends highlight the importance of early interventions, particularly among high-risk male populations, to reduce both immediate transmission and long-term chronic disease burden.

**Fig 4:**
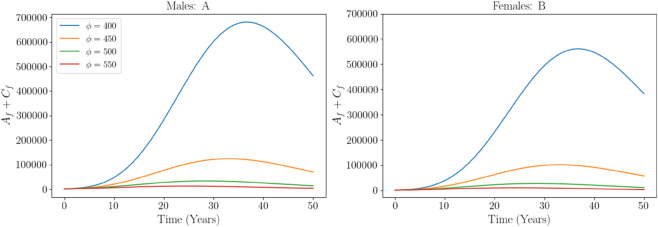
Impact of increasing viral decay rate (*ϕ*) on total acute and chronic HCV infections in males and females over 50 years.

Fig 4 depicts the effect of varying the viral decay rate in contaminated needles (*ϕ*) on the total number of acute and chronic HCV infections in males and females over a 50 year period. In both panels, increasing *ϕ* from 400 to 550 results in a reduction in infections. For males (panel A), the peak infection burden exceeds 650000 individuals at ϕ=400, but drops dramatically as *ϕ* increases, indicating that faster viral clearance from the environment significantly curtails the transmission. A similar pattern is observed in females (panel B), although the overall infection burden is lower. These results highlight the critical role of environmental interventions, such as a focus on needle sterilization and disposal strategies, in reducing HCV transmission. Improving the decay rate of the virus in contaminated needles proves effective for both sexes, supporting the implementation of public health measures that target the injection environment.

**Fig 5:**
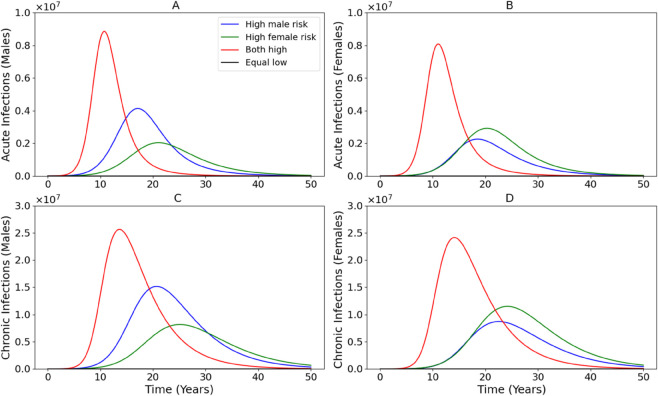
Impact of varying male ($\beta_{m}$) and female ($\beta_{f}$) injection-related transmission rates on HCV dynamics. Panels A and B show acute infections in males and females, respectively, while Panels C and D show chronic infections. Scenarios include high male risk (blue), high female risk (green), both high (red), and equal low risk (black). The scenarios carried out are shown in Table 4.

Fig 5 explores the impact of varying the injection rate times infection of host rate also known as the effective contact rate for males ($\beta_{m}$) and females ($\beta_{f}$) on the spread and progression of HCV. In all panels, the scenario where both $\beta_{m}$ and $\beta_{f}$ are highest (‘Both high,’ red curves) results in the highest peaks for both acute and chronic infections, reflecting rapid transmission and saturation of the susceptible population. When only $\beta_{m}$ is elevated (‘High male risk,’ blue), males experience a greater infection burden (Panels A and C), while the effect on females is modest. The opposite occurs in the ‘High female risk’ scenario (green), where increased $\beta_{f}$ leads to higher infections among females (Panels B and D). This imbalance highlights how risk in one group can still propagate infection in the other due to shared injection environments. The ‘Equal low’ scenario (black), where both $\beta_{m}$ and $\beta_{f}$ are reduced, has the lowest infection levels across all compartments. It is worth stating that the curve is visually negligible due to scale. Compared to scenarios that focus on one sex, reducing risk in both sexes simultaneously leads to the greatest reduction in HCV burden. From a public health standpoint, these results make clear that efforts should not focus solely on men or women, but must instead implement inclusive strategies that address risk behaviors across the entire at-risk population.

**Table 4:** Simulation scenarios with corresponding $\beta_{m}$, $\beta_{f}$ values, and associated plot colors as shown in Fig 5.

Scenario	$\beta_{m}$	$\beta_{f}$	Color
High male risk	1.96854	0.761899	blue
High female risk	0.98427	1.523798	green
Both high	1.96854	1.523798	red
Equal low	0.6	0.6	black

**Fig 6:**
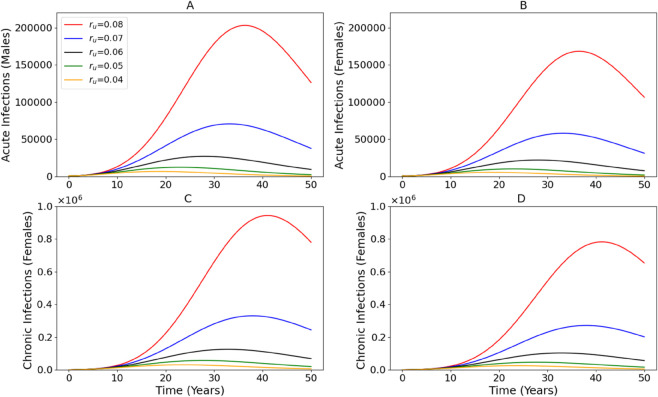
Impact of increasing viral decay rate (*ϕ*) on total acute and chronic HCV infections in males and females over 50 years.

Fig 6 shows the impact of varying the discard rate of uninfected needles (*r*_*u*_) on the progression of HCV infections across sexes and disease stages. As *r*_*u*_ decreases from 0.08 (ineffective needle retention) to 0.04 (strong needle exchange intervention), the burden of infection drops significantly in both sexes and across acute and chronic stages. The most severe outcomes are observed under *r*_*u*_ = 0.08 where peak acute infections reach over 200000 individuals in both males and females, and chronic cases approach 1 million. In contrast, when *r*_*u*_ is reduced to 0.04, peak infections are drastically lower by more than 90% in some cases. This highlights the strong sensitivity of the epidemic to needle handling practices, particularly in environments with shared injection equipment. Comparing the panels, males and females show similar qualitative trends, suggesting that the benefits of needle exchange programs are broadly effective regardless of sex. However, males show slightly earlier and sharper peaks in acute infections, potentially indicating differences in exposure or injection behavior. The results support the expansion and strengthening of needle exchange programs as a key harm reduction strategy. By reducing the discard rate of clean needles, viral transmission through the contaminated needle environment can be drastically curtailed. Such interventions not only reduce new acute infections but also prevent the long-term accumulation of chronic HCV cases. These findings highlight that even modest improvements in needle hygiene can translate into substantial population-level benefits and should be a central component of HCV and broader hepatitis harm reduction policies.

## 5 Discussion

HCV poses a significant global health burden, with the primary mode of transmission in high-income countries being the sharing of contaminated needles among people who inject drugs (PWID). In the USA, HCV remains a major public health concern with ongoing transmission driven largely by the opioid epidemic and injection-related risk behaviors. These sex-specific differences in behavior and outcomes highlight the need for mathematical models that are important in understanding the dynamics, in order to better inform targeted interventions.

To try and understand the transmission dynamics, we developed a sex-structured, environmentally mediated HCV transmission model that includes male and female populations. The model accounts for transmission through contaminated needles, stratifies infections into acute and chronic stages, and incorporates a shared environmental reservoir of infection. We managed to compute the steady states, namely the disease free equilibrium and the endemic equilibrium point. The threshold parameter known as the reproduction number was also computed. The model was calibrated to cumulative case data from the USA, and it demonstrated good agreement with observed trends for both males and females (Fig 1, Panel A). Notably, the model captures the higher cumulative burden of HCV among men, which agrees with known differences in injection-related risk behaviors [59]. The long-term projections (Fig 2, Panel B) suggest that cumulative infections will continue to rise until around 2035 before gradually declining. These findings indicate that, without interventions, both sexes particularly men will continue to experience a growing burden of HCV. Calibration was conducted in Berkeley Madonna, which offers limited statistical diagnostics, therefore, we reported the RMS along with the NRMS, as the RMS appeared large due to the large values we used and the cumulative, exponentially increasing nature of the data. The NRMS provides a scale-independent assessment of fit quality, demonstrating that the model adequately captures the epidemic trend despite numerical inflation in absolute error.

Our sensitivity analysis using PRCCs identified the most influential parameters driving peak acute infections. Among them, the contaminated needle generation rate ($\beta_{c}$), the effective contact rates for both males and females ($\beta_{m}$, $\beta_{f}$), and the male recovery proportion ($\alpha_{m}$) showed strong positive correlations (Fig 2). Conversely, parameters related to environmental viral clearance (*ϕ*) and disease progression ($\theta_{m}$) showed strong negative associations. These results emphasize the importance of both behavioral and environmental dynamics, particularly the need to improve needle sterilization and disposal strategies, and to increase access to recovery and treatment programs tailored to sex-specific needs. It is worth stating that the policy scenarios were implemented as sex-neutral proportional changes to transmission/contact parameters; observed sex differences reflect baseline heterogeneity rather than sex-differential intervention uptake. This points to positive results from suppressing contamination and contacts at the source by expanding low–dead-space syringe distribution, supervised injection with on-site sterilization and disposal, and fast retrieval of discarded syringes to reduce $\beta_{c}$, $\beta_{m}$, and $\beta_{f}$ while increasing *ϕ*. At the same time, faster linkage to care and structured support for men (without limiting women’s access) can reduce the transient peak driven by higher $\alpha_{m}$, even if longer-term gains come later.

Long-term simulation outputs further illustrate the natural progression of HCV infections. Acute infections peak earlier (around year 30) than chronic infections (around year 35–40), with males consistently showing higher burdens in both categories (Fig 3). This reflects both higher initial exposure and faster progression dynamics among men. These trends support the importance of early, sustained interventions targeted toward high-risk populations, mostly men before infections transition into the chronic stage. We also explored the role of environmental interventions by varying the viral decay rate in contaminated needles (*ϕ*). As shown in Fig 4, increasing *ϕ* led to marked reductions in total acute and chronic infections across sexes. These results highlight the importance of environmental hygiene in injection settings and validate needle sterilization as an effective control strategy.

Long-term simulation outputs further illustrate the natural progression of HCV infections. Acute infections peak earlier (around year 30) than chronic infections (around year 35–40), with males consistently showing higher burdens in both categories (Fig 3). This reflects both higher initial exposure and faster progression dynamics among men. These trends support the importance of early, sustained interventions targeted toward high-risk populations, mostly men, before infections transition into the chronic stage. This suggests prioritizing outreach and screening programs in male dominated high-risk settings, such as injection drug use networks, to identify and treat acute cases early, potentially through mobile testing units or community based health campaigns. We also explored the role of environmental interventions by varying the viral decay rate in contaminated needles (*ϕ*). As shown in Fig 4, increasing *ϕ* led to marked reductions in total acute and chronic infections across sexes. These results highlight the importance of environmental hygiene in injection settings and validate needle sterilization as an effective control strategy. This highlights the need for scalable interventions like widespread distribution of sterilization kits, implementation of supervised injection facilities with rigorous cleaning protocols, and public health policies that incentivize proper needle disposal to promote environmental viral clearance and reduce transmission risk.

In addition, we examined how behavioral risk heterogeneity affects epidemic dynamics by varying $\beta_{m}$ and $\beta_{f}$. Fig 5 demonstrates that the highest infection peaks occur when both male and female effective contact rates are elevated, while the lowest infection levels result when both are reduced. The asymmetric impact of increasing either $\beta_{m}$ or $\beta_{f}$ alone also shows that risk in one sex can drive infections in the other due to shared injection environments. This finding demonstrates that it is not sufficient to focus on one sex; instead, comprehensive, inclusive harm reduction strategies targeting all at risk individuals are necessary to meaningfully suppress transmission. Finally, we evaluated the impact of needle exchange programs by varying the discard rate of uninfected needles (*r*_*u*_). Fig 6 shows that decreasing *r*_*u*_ leads to meaningful reductions in acute and chronic infections, further supporting the public health value of improving access to clean injection equipment. These benefits were evident across both sexes, although males exhibited slightly earlier and sharper peaks, potentially reflecting behavioral differences. Importantly, even modest improvements in needle hygiene resulted in dramatic reductions in infection burden, indicating that enhancing needle retention and distribution policies should be a central component of HCV control strategies.

While this study provides important insights into the sex-specific dynamics of HCV transmission and the contribution of behavioral and environmental pathways, it has several limitations. First, the model assumes homogeneous mixing within and between sexes, which may not capture complex social and network based patterns of needle sharing observed in real world settings. Second, the model does not explicitly incorporate age structure. Third, the proportion progressing from acute to chronic infection was assumed to be fixed by sex, whereas in practice it may vary with comorbidities, access to care, and treatment adherence. For future work, we plan to extend the model to include age-specific compartments, varying treatment-access scenarios, and harm-reduction interventions (e.g., opioid substitution therapy and supervised injection services), and to consider explicit DAA control (including impulsive dosing) and a coarse immune/regeneration module [60,61]. Incorporating stochastic elements or network-based approaches could better capture local transmission dynamics and variability, and linking the model to economic evaluation frameworks may support cost-effectiveness analyses of targeted versus population-wide interventions. Explicit treatment effects (e.g., DAAs) were not included, as our aim was to characterize baseline transmission dynamics. Nevertheless, recovery in the present framework may be viewed as recovery, and a dedicated treatment compartment is a natural extension for future work. Finally, as more empirical data become available, particularly post-2020, model refinement and validation will be critical for informing ongoing public health strategies.

### Supporting information

S1 Text All additional methodological derivations are presented in the supplementary materials, proofs (Appendix A), model fit metrics (Appendix B), extended epidemiological data (Appendix C), and summary tables of key findings (Appendix D) are provided in the Supplementary Material. (PDF)
