# Supplementary material for: A mathematical model of HCV transmission dynamics with sex stratification and environmental effects
Source: PLoS One. 2025 Dec 1;20(12):e0336374. doi: 10.1371/journal.pone.0336374 (PMC12668632; doi:10.1371/journal.pone.0336374)
Supplement: S1 Text — (PDF) [file pone.0336374.s001.pdf]

A mathematical model of HCV transmission dynamics with sex stratification and environmental effects

Mlyashimbi Helikumi[1,*], Josiah Mushanyu[2], Adquate Mhlanga[3]

[1] Mbeya University of Science and Technology, College of Science and Technical Education,

Department of Mathematics and Statistics, P.O. Box 131, Mbeya, Tanzania,

[2] Department of Computing, Mathematical & Statistical Science, University of Namibia, Windhoek 13301, Namibia

[3] The Program for Experimental and Theoretical Modeling, Division of Hepatology, Department of Medicine,

Stritch School of Medicine, Loyola University Chicago, Maywood, IL 84101, USA.

Appendix A

This appendix presents a proof of Theorem 1 for model (1).

Proof. We first establish the positivity of solutions for non-negative initial conditions. Define

$t_1 = \sup\{t > 0 : S_i(t) > 0, A_i(t) > 0, C_i(t) > 0, R_i(t) > 0, W_u(t) > 0, W_c(t) > 0 \, (i = m, f) \text{ for } t \in [0, t_1]\}.$

Since the initial conditions are non-negative and the right-hand sides are continuous, $t_1 > 0$.
For S_i $(i = m, f)$:

$$\frac{dS_i}{dt} = \Lambda_i - (\mu_i + \lambda_i)S_i \geq -(\mu_i + \lambda_i)S_i,$$

where $\lambda_i = \beta_i \frac{W_c}{W_u + W_c} \geq 0$. This can be rewritten as

$$\frac{d}{dt}\left(S_i(t)\exp\left\{\int_0^t (\mu_i + \lambda_i(\tau))\,d\tau\right\}\right) \geq \Lambda_i \exp\left\{\int_0^t (\mu_i + \lambda_i(\tau))\,d\tau\right\}.$$

Integrating both sides from 0 to t_1 yields

$$S_i(t_1)\exp\left\{\int_0^{t_1} (\mu_i + \lambda_i(\tau))\,d\tau\right\} - S_i(0) \geq \int_0^{t_1} \Lambda_i \exp\left\{\int_0^{y} (\mu_i + \lambda_i(\tau))\,d\tau\right\} dy,$$

so

$$S_i(t_1) \geq S_i(0)\exp\left\{-\int_0^{t_1} (\mu_i + \lambda_i(\tau))\,d\tau\right\} + \exp\left\{-\int_0^{t_1} (\mu_i + \lambda_i(\tau))\,d\tau\right\}\left(\int_0^{t_1} \Lambda_i \exp\left\{\int_0^{y} (\mu_i + \lambda_i(\tau))\,d\tau\right\} dy\right) > 0,$$

*Correspondence email: :mhelikumi@yahoo.co.uk

since $\Lambda_i > 0$.

For A_i $(i = m, f)$:

$$\frac{dA_i}{dt} = \lambda_i S_i - (\mu_i + \theta_i)A_i \geq -(\mu_i + \theta_i)A_i,$$

as $\lambda_i S_i \geq 0$. Thus,

$$\frac{d}{dt}\left(A_i(t)\exp\{(\mu_i + \theta_i)t\}\right) \geq 0.$$

Integrating from 0 to t_1 gives

$$A_i(t_1)\exp\{(\mu_i + \theta_i)t_1\} \geq A_i(0) > 0,$$

so $A_i(t_1) > 0$.

For C_i $(i = m, f)$:

$$\frac{dC_i}{dt} = \alpha_i \theta_i A_i - (\mu_i + v_i)C_i \geq -(\mu_i + v_i)C_i,$$

yielding $C_i(t_1) > 0$.

For R_i $(i = m, f)$:

$$\frac{dR_i}{dt} = (1 - \alpha_i)\theta_i A_i - \mu_i R_i \geq -\mu_i R_i,$$

yielding $R_i(t_1) > 0$.

For W_u:

$$\frac{dW_u}{dt} = b - (\lambda_c + r_u)W_u + \phi W_c \geq -(\lambda_c + r_u)W_u,$$

yielding $W_u(t_1) > 0$.

For W_c:

$$\frac{dW_c}{dt} = \lambda_c W_u - (\phi + r_c)W_c \geq -(\phi + r_c)W_c,$$

yielding $W_c(t_1) > 0$.

This contradiction implies $t_1 = \infty$, so all solutions remain non-negative for $t > 0$.

Now, we prove boundedness. Let $N_i = S_i + A_i + C_i + R_i$ $(i = m, f)$:

$$\frac{dN_i}{dt} = \Lambda_i - \mu_i N_i - v_i C_i \leq \Lambda_i - \mu_i N_i,$$

since $v_i C_i \geq 0$. The initial value problem $\Theta_i' = \Lambda_i - \mu_i \Theta_i$, with $\Theta_i(0) = N_i(0)$, has the solution

$$\Theta_i(t) = \frac{\Lambda_i}{\mu_i}(1 - e^{-\mu_i t}) + N_i(0)e^{-\mu_i t},$$

and $\lim_{t\to\infty} \Theta_i(t) = \frac{\Lambda_i}{\mu_i}$. Therefore, $N_i(t) \leq \Theta_i(t)$, which implies $\limsup_{t\to\infty} N_i(t) \leq \frac{\Lambda_i}{\mu_i}$. If $N_i(0) > \frac{\Lambda_i}{\mu_i}$, then $\frac{dN_i}{dt} < 0$.

For the environmental compartments, adding the equations for W_u and W_c:

$$\frac{d}{dt}(W_u + W_c) = b - r_u W_u - r_c W_c \leq b - \min(r_u, r_c)(W_u + W_c).$$

The initial value problem $\Theta' = b - \min(r_u, r_c)\Theta$, with $\Theta(0) = W_u(0) + W_c(0)$, has the solution

$$\Theta(t) = \frac{b}{\min(r_u, r_c)}(1 - e^{-\min(r_u, r_c)t}) + \Theta(0)e^{-\min(r_u, r_c)t},$$

and $\lim_{t\to\infty}\Theta(t) = \frac{b}{\min(r_u,r_c)}$. Therefore, $W_u(t) + W_c(t) \leq \Theta(t)$, which implies $\limsup_{t\to\infty}(W_u(t) + W_c(t)) \leq \frac{b}{\min(r_u,r_c)}$. If $W_u + W_c > \frac{b}{\min(r_u,r_c)}$, then $\frac{d}{dt}(W_u + W_c) < 0$.

The right-hand sides of model (1) are locally Lipschitz in $\mathbb{R}^{10}_{+}$, so local solutions exist and are unique. Boundedness prevents finite-time blow-up, implying global existence. Solutions starting in Ω satisfy the inequalities above, remaining in Ω (positive invariance). Solutions starting outside enter Ω asymptotically (attracting), as shown by the limsup bounds and negative derivatives at the boundaries. □

Appendix B

Model Fit Metrics

The model was fitted simultaneously to HCV cases for males and females over the period 2006–2021 using Madonna 10.6.1 [4], which provided a Root Mean Square (RMS) error of 1025.28543. The RMS is calculated as:

$$\text{RMS} = \sqrt{\frac{1}{n}\sum_{i=1}^{n}(y_i - \hat{y}_i)^2} \tag{1}$$

where n is the number of data points, y_i is the observed value, and $\hat{y}_i$ is the predicted value. This large RMS value reflects the scale of the data (ranging from hundreds to tens of thousands of cases), making it difficult to interpret the error's significance directly. A very large RMS can obscure the relative accuracy of the model, potentially misleading about its performance. To address this, the RMS was normalized by the mean of the observed total cases (4936.5), yielding a Normalized Root Mean Square (NRMS):

$$\text{NRMS} = \frac{\text{RMS}}{\text{Mean of Observed Data}} = \frac{\sqrt{\frac{1}{n}\sum_{i=1}^{n}(y_i - \hat{y}_i)^2}}{\frac{1}{n}\sum_{i=1}^{n} y_i} \tag{2}$$

This resulted in an NRMS of approximately 0.2077, indicating that the model's predictions deviate from the observed data by about 20.77% of the mean. This normalization provides a clearer assessment of the fit, independent of the data's scale. The moderate NRMS suggests a reasonable fit to the data.

Appendix C

Year	Total P.	Male %	Female %	Male Cases	Female Cases	Male C.	Female C.
2006	298,379,912	0.3	0.2	895	597	895	597
2007	301,231,207	0.3	0.3	904	904	1,799	1,501
2008	304,093,966	0.3	0.3	912	912	2,711	2,413
2009	306,771,529	0.3	0.3	920	920	3,631	3,333
2010	309,327,143	0.3	0.3	928	928	4,559	4,261
2011	311,583,481	0.4	0.4	1,246	1,246	5,805	5,507
2012	313,877,662	0.7	0.5	2,197	1,569	8,002	7,076
2013	316,059,947	0.8	0.7	2,528	2,212	10,530	9,288
2014	318,386,329	0.8	0.7	2,547	2,229	13,077	11,517
2015	320,738,994	0.9	0.7	2,887	2,245	15,964	13,762
2016	323,071,755	1.1	0.8	3,554	2,585	19,518	16,347
2017	325,122,128	1.2	0.9	3,901	2,926	23,419	19,273
2018	326,838,199	1.3	1.0	4,249	3,268	27,668	22,541
2019	328,329,953	1.6	1.0	5,253	3,283	32,921	25,824
2020	331,526,933	2.0	1.0	6,631	3,315	39,552	29,139
2021	332,048,977	2.1	1.0	6,973	3,320	46,525	32,459

Table 1: Table S2: Annual U.S. population and estimated number of HCV-infected individuals by sex (2006–2021), based on CDC surveillance data [1]. Total population numbers are from U.S. Census Bureau's annual and intercensal estimates [2, 3]. Male and female case counts are calculated as Population × Sex-specific prevalence proportion. Cumulative totals (Male C., Female C.) are year-on-year running sums of annual new cases.

Appendix D

Finding Category	Key Results	Implications for HCV Control
**Gender-Specific Burden**	Males experience higher acute and chronic infection peaks (e.g., acute peaks at ∼year 35–40 for males vs. ∼year 30 for females; males bear a disproportionately higher cumulative burden, as fitted to U.S. data and projected to 2040).	Prioritize targeted interventions for high-risk male PWID, such as enhanced treatment access, while maintaining inclusive strategies for females to address disparities.
**Sensitivity Analysis (PRCC)**	Strong positive correlations: male recovery proportion (α_m), contaminated needle generation rate (β_c), and effective contact rates (β_m, β_f).<br>Strong negative correlations: viral decay rate in needles (ϕ) and progression to chronic infection in males (θ_m).	Focus on reducing needle contamination (β_c) and risky behaviors (β_m, β_f) through education and harm reduction. Promote faster viral clearance (ϕ) via sterilization and improve male recovery (α_m) with gender-tailored programs.
**Impact of Viral Decay Rate (ϕ)**	Increasing ϕ from 400 to 550 reduces total acute/chronic infections (e.g., male peak drops from $> 650,000$ at $\phi = 400$; similar but lower burden in females).	Environmental interventions like needle sterilization and disposal are highly effective for both sexes; implement public health measures targeting injection environments.
**Impact of Risky Behaviors (β_m, β_f)**	Highest peaks when both β_m and β_f are high; lowest when both are reduced (e.g., ”equal low” scenario nearly eliminates visible peaks). Asymmetric effects: high β_m drives more male infections, high β_f drives more female infections, but spillover occurs due to shared environments.	Comprehensive, inclusive strategies reducing risky injection behaviors in both sexes yield the greatest reductions; avoid sex-specific silos and address shared needle use.
**Impact of Needle Exchange (r_u)**	Decreasing r_u from 0.08 to 0.04 reduces infections by $> 90\%$ in some cases (e.g., acute peaks drop from $> 200,000$; benefits across acute/chronic stages and sexes, with males showing earlier/sharper peaks).	Strengthen needle exchange programs to minimize reuse of clean needles; this curbs transmission through contaminated environments and provides broad, sex-agnostic benefits.

Table 2: Table S1: Summary of key model findings and implications for HCV control strategies.

References

[1] U.S. Centers for Disease Control and Prevention. (2022). *Hepatitis C surveillance data – Sex-specific case rates and trends*. In *2022 Viral Hepatitis Surveillance Report*. Retrieved from https://www.cdc.gov/hepatitis-surveillance-2022/hepatitis-c/figure-3-5.html

[2] U.S. Census Bureau. (2021). *Annual estimates of the resident population for the United States, regions, states, the District of Columbia, and Puerto Rico: April 1, 2010 to July 1, 2019; April 1, 2020; and July 1, 2020 (NST-EST2020)* [Data set]. Population Division. Available at: https://www.census.gov/data/tables/time-series/demo/popest/2010s-national-total.html

[3] U.S. Census Bureau. (2012). *Intercensal estimates of the resident population for the United States, regions, states, District of Columbia, and Puerto Rico: April 1, 2000 to July 1, 2010 (NST-EST2012-01)* [Data set]. Population Division. Available at: https://www.census.gov/data/tables/time-series/demo/popest/intercensal-2000-2010-state.html

[4] Madonna 10.6.1 [Computer software]. (n.d.). Berkeley Madonna, Inc. Retrieved from https://www.berkeleymadonna.com/
